# Quinolone Antimicrobial Agents, 3rd Edition

**DOI:** 10.3201/eid1006.040025

**Published:** 2004-06

**Authors:** Gregory J. Anderson

**Affiliations:** * Centers for Disease Control and Prevention, Atlanta, Georgia, USA

**Keywords:** Quinolone Antimicrobial Agents

The quinolone class is one of the more important classes of antimicrobial agents discovered in recent years and one of the most widely used classes of antimicrobial drugs in clinical medicine. Their broad spectrum of activity and pharmacokinetic properties make the quinolone agents ideal for treating a variety of infections. Their clinical importance is further demonstrated by their activity against a wide range of diseases of public health importance such as anthrax, tuberculosis, bacterial pneumonia, and sexually transmitted diseases.

Recent research has provided new data on the agent's structure-function relationships, modes of action, resistance, pharmacokinetics, and drug interactions. The third edition of Quinolone Antimicrobial Agents nicely addresses these advances. The book is organized into four sections, each containing chapters written by leading experts.

Mechanisms and Spectrum of Activity and Resistance, the first major section, explores the basic biology of the quinolone class. The interrelationships between structure, antimicrobial activity, and side effects associated with various side chain positions of the quinolones are discussed here. Pertinent information on the bacterial topoisomerases and DNA gyrases, quinolone binding, DNA/RNA synthesis inhibition, cell death in the absence of protein synthesis, specific mutations within the quinolone resistance–determining region, and mutations that lead to altered access to target enzymes (efflux systems) is also highlighted.

In the next section, Pharmacology, the intricate field of quinolone pharmacokinetics (PK) and pharmacodynamics (PD) is evaluated. Information regarding absorption, distribution, metabolism, and excretion in a range of patient types is provided, followed by data gathered from pharmacokinetic/pharmacodynamic studies in a variety of models. This section also provides a review of the role of pharmacologic evaluation in optimizing therapy. The last two sections of the book, Clinical Applications and Adverse and Other Effects, will be of particular interest to clinicians. The chapters in this section include a comprehensive reference on general considerations, antimicrobial aspects, treatment models, clinical and comparative studies, and suggested treatment regimens for a host of infections. The chapter on adverse effects has been greatly expanded from the second edition because a substantial body of new information has since been gathered. Topics covered here include allergic reactions, effects on connective tissue structures and pregnancy, phototoxicity, and central nervous system and immune system toxicity.

The organization and content of this text make it a superlative reference. The discussions on treatments and adverse effects contain some of the most current data available. The field of antimicrobial resistance, however, is one of the most rapidly evolving areas with constant discoveries of new cases and data. Users should understand that while this text serves as a valuable reference, other sources should be consulted to ensure that the most comprehensive data are obtained. The references provided throughout, however, give the reader starting points to other literature.

